# Exploring the Impact of a Persuasive Serious Video Game (Farmily) on Promoting Home Gardening Among Novices: Design and Randomized Controlled Trial

**DOI:** 10.2196/60771

**Published:** 2024-08-08

**Authors:** Carlos Alberto García de Alba-Chávez, Ismael Edrein Espinosa-Curiel, Rosa María Michel-Nava

**Affiliations:** 1 Departamento de Sistemas y Computación Instituto Tecnológico de Ciudad Guzmán Tecnológico Nacional de México Ciudad Guzmán, Jalisco Mexico; 2 Unidad de Transferencia Tecnológica Tepic Centro de Investigación Científica y de Educación Superior de Ensenada Tepic, Nayarit Mexico

**Keywords:** serious video game, persuasive game, home gardening, knowledge, attitude, self-efficacy, intention

## Abstract

**Background:**

Home gardens worldwide provide sustenance, economic support, and access to fresh produce and promote household well-being, health, self-sufficiency, and food security. However, they face significant challenges worldwide and necessitate innovative promotion approaches. Serious video games have proven effective in promoting agricultural knowledge. However, more research is needed on the persuasive potential of agriculture games to influence players’ thoughts, attitudes, and behaviors. This provides an opportunity to examine the impact of persuasive games on promoting home gardening among novices.

**Objective:**

This study aims to describe the design and development of Farmily, a persuasive video game promoting home gardening among novices. In addition, it evaluated the effectiveness of Farmily and compared its impact with that of a traditional home gardening workshop. Furthermore, the study explored how game enjoyment relates to the game’s outcomes.

**Methods:**

A randomized controlled trial with 50 novice gardening participants aged 20 to 50 years was carried out. Participants were randomly assigned to a control group (1.5-hour workshop) or an experimental group (1.5-hour Farmily session). Pre- and postintervention assessments were conducted. We evaluated Farmily’s impact on knowledge, attitudes, perceived self-efficacy, and intentions regarding initiating home gardens. In addition, we investigated the user enjoyment and its relationship with the game’s effect outcomes.

**Results:**

The experimental group significantly improved their knowledge (t_24_=4.26; *P*<.001), attitude (*z*_24_=2.98; *P*=.003), self-efficacy (t_24_=2.6; *P*=.02), and intention to initiate home gardens (*z*_24_=4.15; *P*<.001). The experimental group showed similar effectiveness in knowledge transfer (t_24_=–1.71; *P*=.09) and a more significant impact on attitude (*z*_24_=2.73; *P*=.006), self-efficacy (t_24_=2.21; *P*=.03), and intention to start a home garden (t_24_=–5.33; *P*<.001) than the control group. Farmily was well received by the intervention group, generating high enjoyment. Furthermore, user enjoyment substantially correlated with user attitudes (*r*_23_=0.72; *P*<.001) and self-efficacy (*r*_23_=0.67; *P*<.001), yet no discernible association was observed among user enjoyment, knowledge (*r*_23_=0.26; *P*=.20), and intention (*r*_23_=0.06; *P*=.77).

**Conclusions:**

Evidence indicates that Farmily appears to be a viable tool for promoting home gardening among novices in the short term. Farmily demonstrated similar effects in knowledge improvement to those of a traditional workshop and had a more significant impact on the other variables. In addition, we found that the player’s gaming experience positively relates to the player’s attitudes and self-efficacy. A well-powered randomized controlled trial with more diverse samples and extended follow-up periods will be conducted to establish the long-term efficacy of Farmily and gain a deeper understanding of the influence of enjoyment on game outcomes.

## Introduction

### Promotion of Home Gardening

Home gardens, a long-standing tradition of small-scale food cultivation near human settlements [1], remain a cornerstone of agriculture, providing essential sustenance and economic support for families worldwide [2]. These gardens, cultivated for centuries, serve multifaceted purposes within local food systems. Alongside economic benefits such as financial savings and income generation, they provide year-round nutrition and access to fresh produce and contribute to biodiversity conservation [2-4]. In addition, home gardens contribute to ecological production, enhance esthetics, and promote sustainable urban development [5]. Moreover, maintaining and using home gardens contributes significantly to household well-being by promoting psychological and physical health and fostering familiar and social connections and bonds [6]. Another significant benefit is fostering self-sufficiency as individuals cultivate plants or food crops at home to meet their sustenance needs, thereby reducing dependence on external sources [7]. This characteristic plays a crucial role in bolstering food security [8], a role that was particularly emphasized during the COVID-19 pandemic [9]. Due to these advantages, home gardening initiatives have expanded to include elementary schools [10], urban areas, and household environments [11]. They remain a valuable pursuit even for economically active individuals who can afford groceries.

Despite the global significance of home gardens, many people are not interested or hesitant to start one [12], contributing to their decline [13], a trend particularly concerning in countries such as Mexico [14]. Various strategies have been proposed to promote home gardening, ranging from campaigns highlighting its benefits to educational initiatives offering guidance through manuals, courses, workshops, and guides [15]. However, these initiatives often focus on individuals already motivated to start gardening, potentially missing the chance to inspire and engage newcomers. Furthermore, these strategies encounter challenges inherent to the multifaceted nature of home gardening, including the necessity to develop skills; address concerns; overcome motivation issues [16,17]; acquire interdisciplinary knowledge in fields such as biology, chemistry, and economics [10]; and achieve a balance between theoretical learning and hands-on activities [18].

To bridge the gap between promoting home gardening and its adoption, innovative strategies are essential to attract a diverse audience, motivate and empower individuals, and provide practical learning opportunities. It is crucial that these strategies mainly focus on novices—individuals who are open to learning about gardening but may not be strongly motivated to pursue it actively. These individuals often lack extensive knowledge, experience, and confidence in gardening and may not fully grasp its benefits. This situation presents an opportunity to develop accessible and engaging tools that inspire, educate, build confidence, and foster sustained interest in home gardening. Persuasive video games provide a promising platform to enhance users’ knowledge, skills, and experiences through interactive platforms [19] and foster positive changes in perceptions, attitudes, or behaviors [20,21].

### Persuasive Video Games

Persuasive video games, also known as serious games for change or persuasive games, aim to actively engage players while promoting positive changes in their thoughts, attitudes, feelings, actions, or behaviors [20-22]. They integrate interactive gameplay, narrative elements, educational content, and persuasive strategies to foster attitude and behavior change. Despite their serious intent, these games prioritize enjoyment and engagement, leveraging the immersive nature of video games to captivate players and sustain their interest [23]. They often include informative content relevant to the issue, offering players opportunities for learning and reflection during gameplay [24]. These games may use procedural rhetoric through interactive gameplay dynamics to convey persuasive messages effectively [22] and can use exocentric or endocentric approaches to engage and motivate players [24]. Successful play encourages players to develop a deeper understanding beyond the game, potentially influencing their behavior afterward. However, the effectiveness of these games hinges on game features, player characteristics, use context [25], and the balance of persuasive strategies [21].

Persuasive video games are a powerful tool for driving social change [26]. They are studied extensively for their ability to promote attitude and behavior changes across various issues such as politics, society, environment, and health [21]. They address social problems such as attitudes toward homelessness [27], humanitarian aid willingness [28], and workloads [20] while also tackling health issues such as physical activity, nutrition, and disease management [21]. Specific games target smoking cessation [29] and medication adherence [30]. Despite their widespread application, research shows mixed results on their impact, with some studies confirming effectiveness and others not, leading to inconsistent findings [20,21,31]. A systematic review indicated positive or partially positive outcomes [21], but effectiveness varied across studies, underscoring the need for rigorous evaluation across diverse contexts and user profiles [20]. Current research often focuses on overall impacts rather than specific game or user characteristics [31], necessitating further studies on how game features affect effectiveness, especially the effect of user enjoyment [32].

### Persuasive Video Games for Agriculture

Most serious video games for agriculture have been designed as educational tools [33]. Explicitly designed persuasive games are scarce; only a few studies have evaluated their persuasive effects. The educational games cover various aspects of agriculture and vegetable cultivation, including genres such as role-playing games and farm simulators. Examples include “Farmtasia” [34], “Little Botany” [35], “Herbopolis” [36], and Serious Game for Agroecology Learning [37]. They offer valuable learning experiences in farming practices, sustainable agriculture, herbal medicine agriculture, and ecological awareness, leading to knowledge enhancement. However, there need to be more games specifically focusing on home gardening [33]. Another group of studies aims to raise awareness of agriculture-related issues. “AgriVillage” [38] focuses on environmental concerns in agriculture, including the effects of fertilizers and deforestation on water sources and weather patterns. “RebEarth” [39] promotes awareness of hydroponics. Furthermore, individuals who are agricultural novices are often overlooked by current serious video games for agriculture [33].

Among the studies evaluating the persuasive effects of serious agricultural games, an example is “Game of Piglets” [40], a virtual pig farm that allows students to practice external biosecurity and farrowing management procedures. This simulation and adventure game emphasizes core competencies such as farrowing aid, identifying unwell sows after farrowing, maintaining aseptic conditions during surgeries, and ensuring an optimal piglet environment. These competencies are developed through tasks closely resembling real-life scenarios in pig farms. Evaluation results indicated improved perceived self-efficacy among players. Another instance is “MahindiMaster” [41], a serious game simulating crop yields based on farmers’ choices from various input options. These yields are customized using crop model outputs leveraging plot-level soil samples and historical weather data. The game allows farmers to experiment with 3 different fertilizers. Evaluation outcomes revealed positive shifts in players’ beliefs and fertilizer use on their crops. While the evaluation results indicate positive impacts and the viability of using agriculture-focused serious video games to change players’ attitudes and behaviors, the persuasive potential of agricultural video games remains unexplored. It prompts questions about their effectiveness in promoting home gardening among novices.

Inspired by the need for innovative strategies to promote home gardening among novices and recognizing the potential of persuasive video games to influence user knowledge, attitudes, and behaviors, this study addressed existing research gaps in persuasive serious games for home gardening. In total, 3 primary research questions guided our investigation. The first 2 questions focused on the effect of a persuasive game on promoting home gardening among novices:

- Can a persuasive video game encourage novices to engage in home gardening?
- Does a persuasive video game have a more significant impact on changing novices’ knowledge, attitudes, self-efficacy, and intentions than a traditional course?

In addition, our investigation was guided by the following primary research question that sought to understand how the enjoyment derived from the game influenced its effectiveness:

- How does the enjoyment experienced by players of an agriculture-focused persuasive video game relate to the game’s effects?

### Objectives and Hypotheses

This study aimed to design and develop a persuasive video game promoting home gardening among novices called Farmily (Farm+Family) and evaluate its effects.

On the basis of the potential of persuasive video games to promote home gardening, we formulated the hypotheses that Farmily players would exhibit superior outcomes in the following areas after playing (hypothesis 1): home gardening knowledge (hypothesis 1A), attitudes toward home gardens (hypothesis 1B), self-efficacy in home gardening (hypothesis 1C), and intention to start a home garden (hypothesis 1D).

In addition, based on the idea that a persuasive video game offers more effective elements for promoting home gardening among novices compared to a traditional course, we established the hypotheses that Farmily players would demonstrate superior outcomes compared to traditional course attendees in the following areas (hypothesis 2): home gardening knowledge (hypothesis 2A), attitudes toward home gardens (hypothesis 2B), self-efficacy in home gardening (hypothesis 2C), and intention to start a home garden (hypothesis 2D).

Finally, based on the premise that game features, particularly user enjoyment, significantly relate to game outcomes, we formulated the hypotheses that the player’s gaming enjoyment is positively related to (hypothesis 3) home gardening knowledge (hypothesis 3A), attitudes toward home gardens (hypothesis 3B), self-efficacy in home gardening (hypothesis 3C), and intention to start a home garden (hypothesis 3D).

## Methods

### Study Design

This study was divided into 2 main parts. The first part focused on designing and developing the video game Farmily. The second part involved conducting a randomized controlled trial (RCT) to assess the impact of Farmily and validate the formulated hypotheses. In the following sections, we provide a detailed description of the Farmily video game.

### Farmily Video Game

#### Design and Development

**Figure 1:**
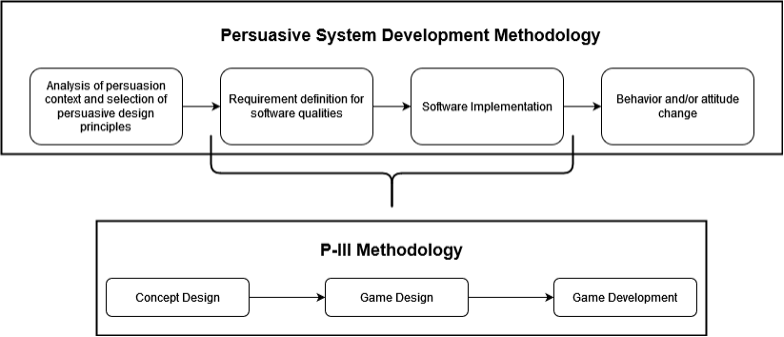
Methodology for the design and development of Farmily. P-III: player-centered, iterative, interdisciplinary, and integrated.

To create Farmily, we integrated 2 methodologies (Figure 1): the persuasive system design (PSD) methodology [42] and the player-centered, iterative, interdisciplinary, and integrated (P-III) methodology [43]. The PSD methodology guides the design of persuasive systems [42], and accordingly, we followed four key steps aligned with persuasive principles. In step 1, we analyzed the context of persuasion and selected appropriate principles and techniques for Farmily. Steps 2 and 3 focused on defining and developing the game, whereas step 4 involved experimentation to evaluate its effectiveness.

During steps 2 and 3 of PSD, given the need to develop a specific persuasive system—a persuasive video game—we used the P-III methodology [43] tailored for serious game design. The P-III methodology emphasizes player-centered design, iterative development, and interdisciplinary collaboration. Following the P-III methodology, we engaged experts in human-computer interaction, agriculture, horticulture, education, and software engineering. In stage 1, the concept design phase, extensive research on home gardening informed the definition of user tasks through co-design sessions and expert consultations. Progressing to stage 2, the video game design phase, we used storyboards, focus groups, and paper prototypes, refining the game iteratively based on continuous feedback. Stage 3, the development phase, involved iterative prototyping and testing. We conducted 21 work sessions, incorporating multiple refinement cycles and expert testing sessions.

#### Theoretical Base and Persuasive Techniques

Farmily is based on the social cognitive theory by Bandura [44,45], which provides valuable insights into individual learning, development, and behavior maintenance in social contexts. This theory highlights the dynamic interplay of personal factors (cognition, beliefs, skills, and affect), environmental factors (social norms, institutions, and cultural influences), and behavioral factors (observable actions and responses) [44,45]. This theory proposes that individuals shape their environments through behavior, and vice versa, environments influence behavior and cognition. Learning, as per this theory, happens through direct experience and observing others [45]. Actions and outcomes offer crucial feedback on behavior appropriateness, with rewards encouraging desirable behaviors and punishments discouraging them. Furthermore, this theory emphasizes self-regulatory processes, which involve activating and sustaining behaviors, cognitions, and emotions directed toward achieving goals. It also highlights various motivational processes, including goals, progress evaluations, outcome expectations, values, and social comparisons. A key concept in the theory by Bandura [44,45] is self-efficacy, which pertains to one’s belief in their capacity to achieve desired outcomes through specific actions. Self-efficacy significantly impacts motivation, goal setting, and persistence, with individuals who possess high self-efficacy more inclined to pursue challenging objectives and persist despite obstacles.

Drawing from the social cognitive theory by Bandura [44,45] and the PSD principles by Oinas-Kukkonen and Harjumaa [42] and Fogg [46], Farmily integrates persuasion techniques. Farmily’s persuasion techniques are classified into primary support, dialogue support, credibility support, and social support, as detailed in Table 1. These strategies aim to engage players’ intellect and motivation, guide behavior, and encourage their interest in starting home gardens. They have proven effective in persuasive video games such as “PowerHouse” [47] and “Smoke?” [21,48].

**Table 1:** Persuasive techniques of Farmily.

Name			Description
**Primary support techniques**			
	Reduction	The game incorporates a virtual environment for family garden ownership and management that simplifies home garden maintenance.	
	Tunneling	The game offers a structured progression system with increasing complexity across levels, introducing new variables for users to understand.	
	Tailoring	Tailored knowledge by dividing levels into 3 difficulty tiers: easy, medium, and hard. Users unlock progressively more advanced and comprehensive information tailored to their learning needs as they advance.	
	Self-monitoring	The score and recommendation system helps players monitor their performance and receive guidance for improvement. This tangible evidence motivates them to persist in learning and practicing.	
	Simulation	Includes a home garden simulation mirroring real-life scenarios, helping players grasp cause-effect relationships between their actions and virtual outcomes.	
	Rehearsal	Provides a platform for simulating various home garden–related behaviors and activities. Through repetitive practice and self-monitoring, players enhance their self-efficacy and proficiency in gardening skills.	
**Dialogue support techniques**			
	Praise	Implements a praise system that offers positive reinforcement to players upon completing tasks or levels. Through motivational messages and rewards, the game aims to encourage continued gameplay.	
	Rewards	Incorporates rewards and motivators such as unlockable content and points to encourage continued play. For instance, users earn a trophy for completing the story mode and stars for each level, enhancing motivation and engagement.	
	Suggestion	The in-game recommendation system guides players in enhancing their gardening performance and achieving higher scores. Tailored suggestions empower players to improve and meet game objectives, boosting their self-efficacy and intention to apply gained knowledge to real-world gardening.	
	Liking	Meticulously recreates a home garden’s visual and sensory experience, immersing players in a realistic virtual environment. This attention to detail enhances players’ liking and connection to the game.	
**Credibility support techniques**			
	Trustworthiness	The game’s gardening knowledge base is built on accurate and authentic information from reliable sources, such as books and manuals recognized and developed by Mexico’s agriculture department and scientific papers sourced from reliable journals, ensuring its trustworthiness and reliability.	
	Surface credibility	The game’s visual design and user interface are meticulously crafted to offer players a professional and credible experience.	
**Social support**			
	Social comparison	Including a leaderboard in the video game enables players to compare their scores and monitor the progress of other users. This feature fosters a competitive and social atmosphere that encourages engagement and interaction among players.	
	Competition	The video game allows players to compete against each other, striving to reach the top of the leaderboard and achieve the highest score. This competitive element enhances motivation and drives players to improve their performance.	
	Recognition	The game recognizes the top 5 players through the leaderboard, highlighting their achievements and establishing a sense of accomplishment. This acknowledgment rewards players and motivates them to continue their efforts and aim for higher rankings.	

#### Description

Farmily is a 3D simulation single-player game available on desktop and Android platforms. It was developed using the Unity game engine (version 2021.3.8f1; Unity Technologies). The players guide and assist a virtual family in managing a home garden. The target users are individuals aged between 20 and 50 years who are novices in home gardening, chosen for suitability. This age range aligns with Mexico’s economically active population (ages of 15 to 64 years) [49] and the gaming demographic [50], capturing a significant portion of gamers interested in home gardening. Variations in the age range do not significantly impact the study’s relevant aspects. The game aims to convey messages such as gaining knowledge about home gardening, recognizing its importance and the enjoyment it generates for individuals and their families, understanding commitments and trade-offs, realizing accessibility and rewards, and acknowledging the multidisciplinary knowledge and risks involved.

**Figure 2:**
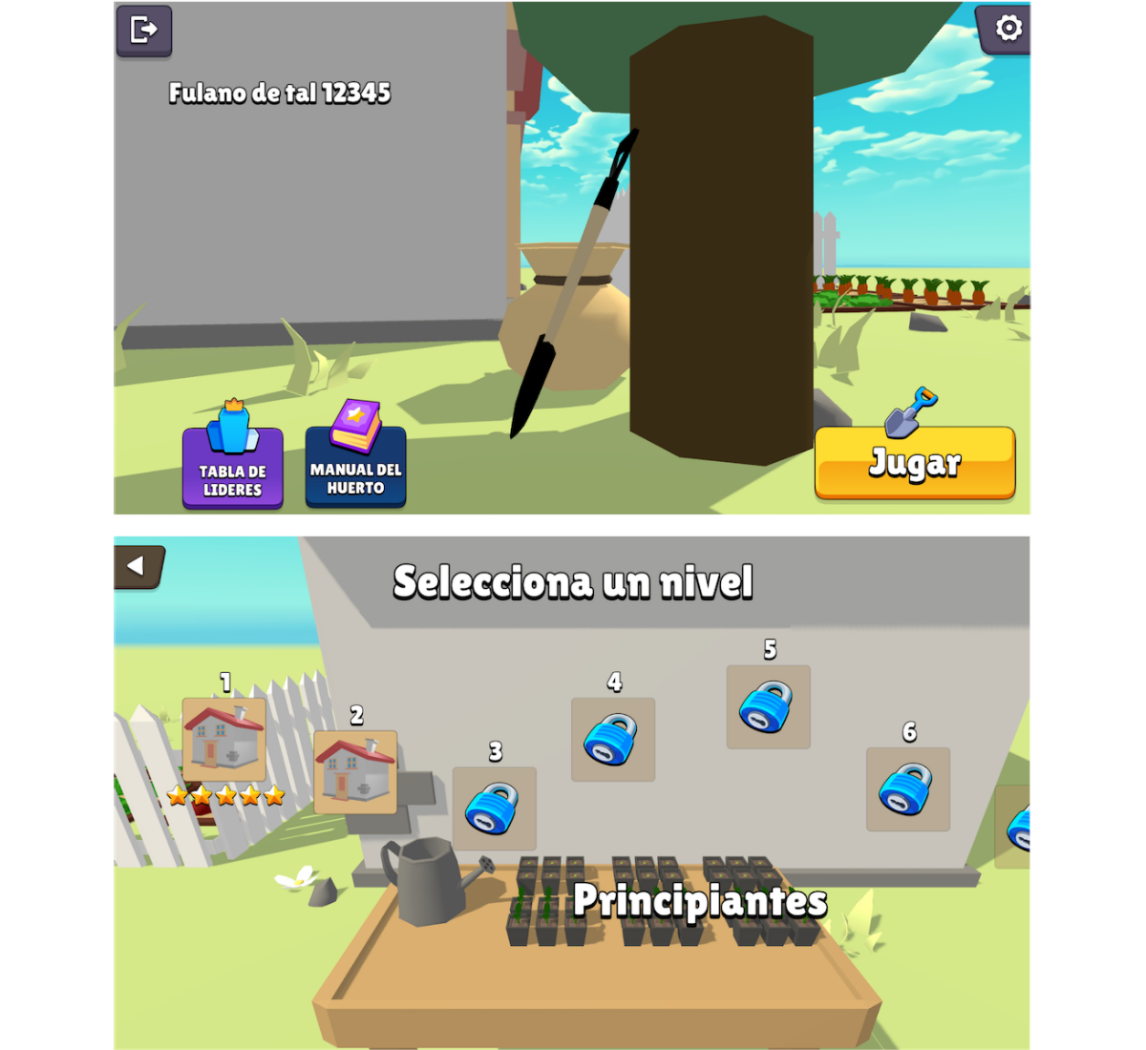
Screenshots of the main menu (top) and level selection (bottom). The game is presented in Spanish.

**Figure 3:**
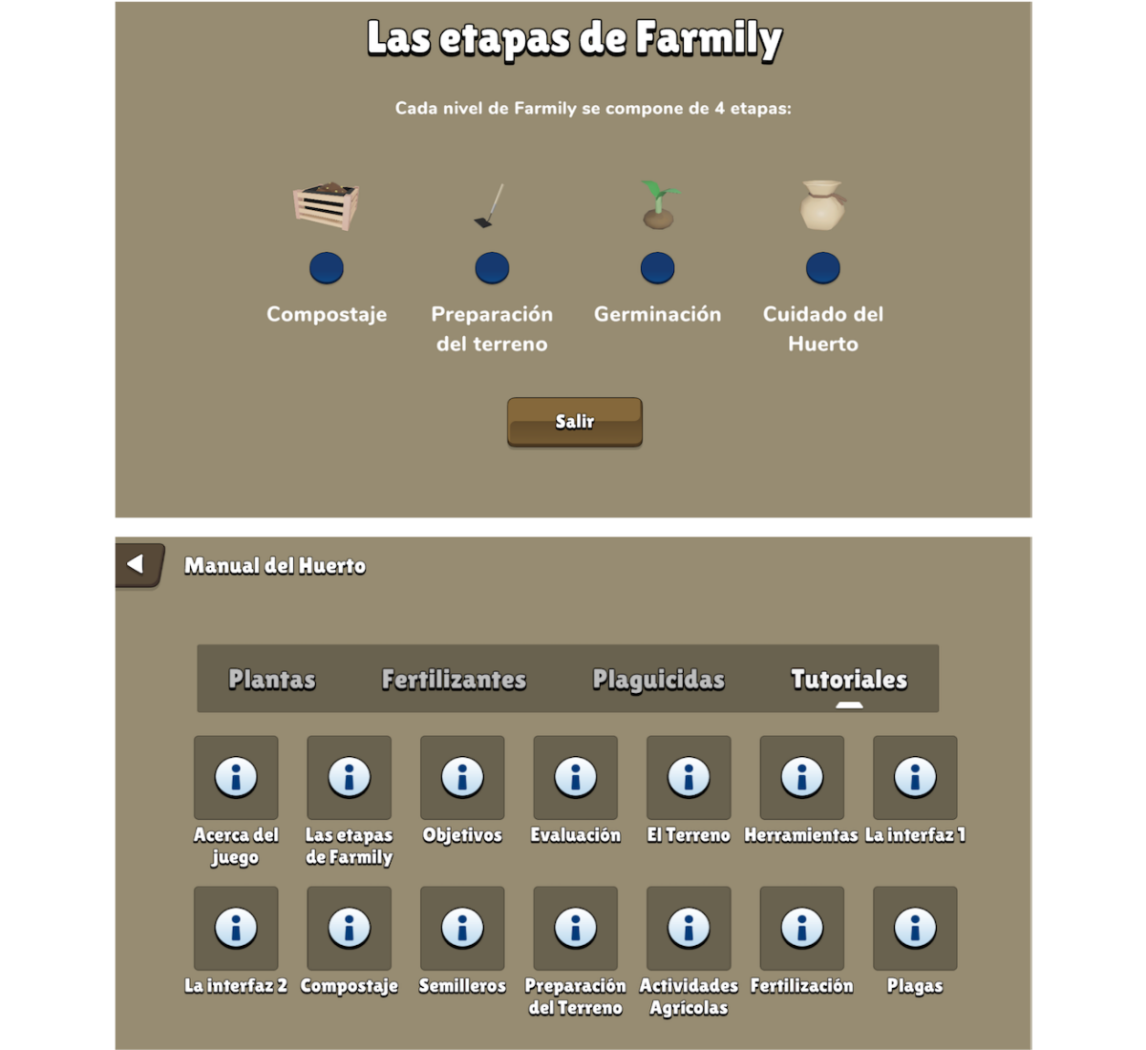
Screenshots of the education section; home gardening phases (top) and tutorial menu (bottom). The game is presented in Spanish.

**Figure 4:**
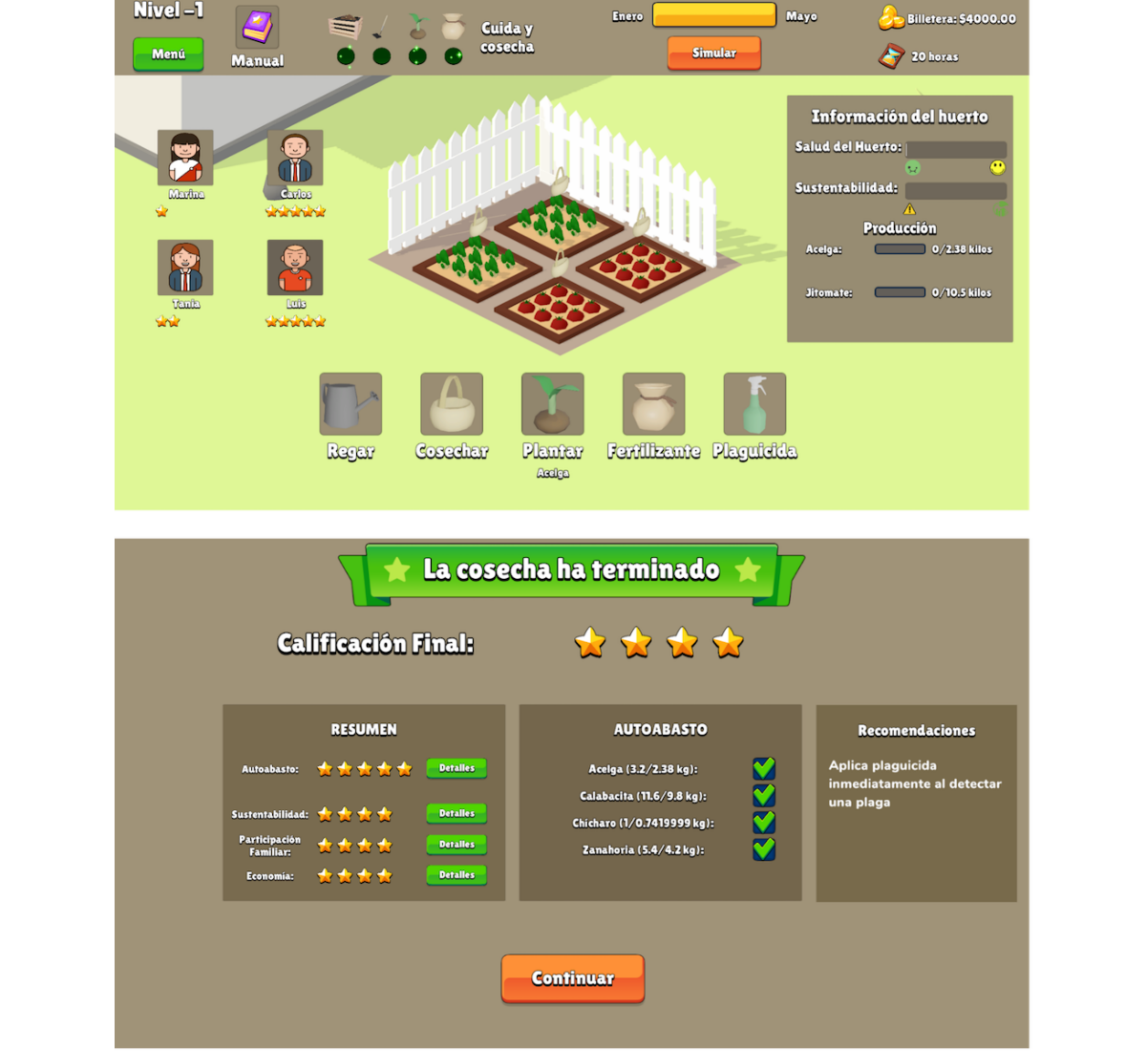
Screenshots of the simulation section; cultivation (top) and level evaluation with recommendations (bottom). The game is presented in Spanish.

Farmily consists of 3 sections: the “Main menu” (Figure 2), “Education” (Figure 3), and “Simulation” (Figure 4). In the main menu, players can register, access the leaderboard, adjust sound settings, and start a gaming session. The educational section is seamlessly integrated into the gameplay, offering tutorials on game mechanics and guidance on executing home gardening tasks both in the game and in real life. The simulation section encompasses the home garden simulation and all associated gameplay mechanics.

The game’s difficulty gradually increases to enhance its educational and persuasive aspects. Farmily offers 20 levels divided into beginner (levels 1-8), medium (levels 9-14), and advanced (levels 15-20) tiers (Figure 2, bottom). Each tier presents challenges in crop yield, finances, and simulation complexity. The level goals include growing and harvesting crops, ensuring family participation, practicing sustainability, maximizing savings, and providing a comprehensive understanding of home gardening implications and benefits. The game educates players on 12 recommended crops for daily consumption [51], empowering informed decisions and gardening optimization. In Farmily, each level follows a systematic progression—players start by preparing compost, clearing undergrowth, and watering and then plow an area spanning 1 to 40 m^2^. Next, they plant seeds in nurseries, nurture them, and transplant them. Subsequently, players water, fertilize, and apply pesticides as needed to ensure crop health for harvesting.

The game provides indicators for garden health, including a progress bar for the current month and phase, meters for overall garden health and sustainability, and progress bars for vegetable quantity (Figure 4, top). Detailed plot information, such as humidity and macronutrient levels, is also available. After harvesting all crops, players receive a rating from 1 to 5 stars based on factors such as harvested vegetables, family involvement, sustainable practices, financial economy, and self-sufficiency (Figure 4, bottom). These ratings unlock new scenarios, motivating players to improve and enhancing replay value. A home garden manual is a central hub for information on crops, fertilizers, pesticides, and game mechanics tutorials (Figure 3, top). Personalized recommendations based on player actions help optimize gardening strategies (Figure 4, bottom). A leaderboard fosters competitive excitement by allowing players to compare scores and aim for the top spot (Figure 2, top).

### Evaluation

#### Overview

In 2023, an RCT was conducted at a Mexican university, with written permission from the school administrators to conduct the trial at the school facilities.

#### Participant Recruitment

We used a convenience sampling approach by inviting 180 students from 9 academic groups to participate based on their availability and interest in the study. Participants were invited without previous information about their backgrounds or characteristics, ensuring a diverse representation across different scholar groups. On the day of the experiment, we verified that participants who agreed to take part met the inclusion criteria, which were (1) being aged between 20 and 50 years; (2) belonging to a family nucleus comprising at least 2 members; (3) having an interest in home gardening; (4) residing in Ciudad Guzmán, Jalisco, Mexico; and (5) having essential elementary-level reading and writing skills in Spanish. Exclusion criteria were applied to individuals who (1) currently or previously had a home garden, (2) had undergone a home gardening course, and (3) had vision or motor impairments that hindered their use of the video game on a PC.

#### Structure and Procedure

Individual participants were randomly assigned to either the control group (CG) or the experimental group (EG). Before the intervention (T_0_), both groups completed 2 pretest questionnaires measuring their knowledge, attitude, self-efficacy, and intention to start a home garden (see the *Measured Variables* section). During the intervention, participants in the CG engaged in a 1.5-hour home gardening workshop facilitated by an expert in agriculture. The workshop provided participants with the same valuable information regarding the benefits, requirements, and activities associated with maintaining a home garden as that provided in the EG and Farmily. The expert presented the knowledge in a lecture format without hands-on experience with a home garden or interactive activities. This workshop mirrors the traditional way of fostering home gardening. In contrast, participants in the EG played Farmily for 1.5 hours. Finally, after the intervention period (T_1_), the CG and EG participants completed the same questionnaires applied during the pretest. This posttest measurement served as a valuable indicator of any changes or improvements in these domains following the intervention. In addition to the posttest questionnaires, participants in the EG were also asked to provide feedback on their enjoyment of the video game using a questionnaire.

#### Measured Variables

The following variables were measured before (T_0_) and after (T_1_) the intervention: (1) knowledge about home gardening, (2) attitude toward home gardening, (3) self-efficacy in home gardening, and (4) intention to start a home garden. In addition, in T_1_, we measured the users’ enjoyment. Textbox 1 describes the measured variables. In the following paragraphs, we describe the methods used for their measurement.

**Description of the measured variables.**

**Knowledge about home gardening**

- This is the comprehension level of a home garden’s concepts and procedures.

**Attitude toward home gardening**

- Fishbein and Ajzen [52] define attitude as “an individual’s positive or negative feelings towards realizing an objective behavior.” In line with this, we defined *attitude toward home gardening* as “an individual’s positive or negative thoughts about having a home garden.”

**Self-efficacy on home gardens**

- Self-efficacy is “people’s beliefs about their abilities to produce a certain level of performance that influences events that affect their lives” [53]. Given this concept, *self-efficacy in home gardening* was defined as “people’s beliefs about their abilities to have a home garden with a certain level of performance that influences the events that affect their lives.”

**Intention to start a home garden**

- Intention is “a person’s motivation in the sense of their conscious plan or decision to exert effort to perform a behavior” [52]. On the basis of this concept, *intention to start a home garden* was defined as “the conscious motivation of a person to start a home garden.”

**Game enjoyment**

- It is defined as “a person’s perception and response as a result of using or anticipating using a product, system, or service” [54].

To evaluate participants’ knowledge levels effectively, we developed a 23-item questionnaire with a home gardening expert, ensuring accuracy and relevance. The questionnaire items were strategically structured into 3 difficulty levels (easy, medium, and hard) designed to assess participants’ understanding comprehensively. Each item provided 4 answer options, with only 1 correct answer. For example, sample questions included an easy query, such as “How many days does it take for a plant to germinate?”; a medium-level question, such as “Which of the following statements best describes the advantages of sustainable products?”; and a hard question, such as “What is a critical factor for achieving success in home gardening?”

To assess attitudes toward home gardens, self-efficacy in home gardening, and the intention to start a home garden, we developed a 14-item questionnaire. This questionnaire draws upon the attitude, self-efficacy, and intention scales from the Unified Theory of Acceptance and Use of Technology (UTAUT) [55]. The UTAUT questionnaire’s validity has been established through rigorous testing and has been shown to account for 70% of the variance in intention [55]. Furthermore, the scale’s reliability was demonstrated with a Cronbach α of 0.70 across all scales [55]. The attitude toward home gardens scale comprises 4 items, the self-efficacy scale includes 7 items, and the intention to start a home garden scale consists of 3 items. Each item is rated on a 5-point Likert scale ranging from “1—Totally disagree” to “5—Totally agree.” Sample questions include “Are home gardens a beneficial idea?” (attitude toward home gardens), “Compared to other people, can I perform my home gardening activities correctly?” (self-efficacy), and “I plan to start a home garden in the next six months.” (intention to start a home garden).

We used the EGameFlow questionnaire [56], a validated tool of 42 items, to assess user enjoyment. Comprehensive testing confirmed the questionnaire’s validity, which was robust enough to account for 74% of the variance in learner enjoyment [56]. Moreover, the EGameFlow questionnaire exhibited strong reliability, boasting a Cronbach α of 0.942, signifying excellent internal consistency. In addition, it demonstrated good test-retest reliability [56]. The questionnaire items are categorized into 8 dimensions: concentration (6 items), clear goal (4 items), feedback (5 items), challenge (6 items), autonomy (3 items), immersion (7 items), social interaction (6 items), and knowledge improvement (5 items). Each item is rated on a 5-point Likert scale ranging from “1—Totally disagree” to “5—Totally agree.”

#### Data Analysis

The data analysis was performed using the R software (version 4.3.0; R Foundation for Statistical Computing) on the Windows platform. A significance level of 5% (*P*<.05) was used to determine statistical significance. The normality of the numerical data was assessed using the Shapiro-Wilk test. Parametric tests were used for normally distributed data, while nonparametric tests were used for nonnormal data. Changes in knowledge within the same group were analyzed using a 1-tailed paired *t* test. A 2-tailed independent-sample *t* test was conducted to compare knowledge between groups. The Wilcoxon signed rank test was applied for questions with ordinal values, such as attitude, self-efficacy, intention, and user enjoyment. The 2-tailed paired *t* test was used to analyze differences from T_0_ to T_1_ in ordinal question scales within the same group assuming that the data followed a normal distribution. Alternatively, the Wilcoxon signed rank test was used. For comparisons between groups and when the data followed a normal distribution, a 2-tailed independent-sample *t* test was used. In the absence of normal distribution, a Mann-Whitney *U* test was conducted. The internal consistency of the user enjoyment scales was assessed using the Cronbach α. Finally, the bilateral Pearson correlation test was conducted to examine the relationships between user enjoyment and posttest scores on knowledge, attitude, self-efficacy, and intention to start a home garden.

### Ethical Considerations

The institutional bioethics review board of the Centro de Investigación Científica y de Educación Superior de Ensenada approved all study procedures (BIOETICA_HUM_2023_03). Participants who expressed interest in joining the study provided written informed consent. The study ensured that all collected data were anonymized or de-identified to protect personal information, implementing strict protective measures for any data that could not be fully anonymized. Participation in the study was voluntary and uncompensated.

## Results

### Participant Characteristics

Of the 180 students and teachers approached, 50 (27.8%) agreed to participate in the study. The participants had an average age of 21.5 (SD 6.8) years, with 66% (33/50) being male and 34% (17/50) being female. Among the participants, the EG consisted of 25 individuals with an average age of 20.2 (SD 0.7) years, comprising 11 (44%) female and 14 (56%) male individuals. The CG also consisted of 25 participants with an average age of 22.7 (SD 9.5) years, including 6 (24%) female and 19 (76%) male individuals.

### Knowledge of Home Gardening

The knowledge of participants (Table 2) in the EG significantly increased from T_0_ to T_1_ (mean difference 3.2, SD 0.18; t_24_=4.26; *P*<.001). Similarly, the CG showed a significant increase in knowledge (mean difference 5, SD 0.3; t_24_=6.78; *P*<.001). Comparing the differences in knowledge increases between groups from T_0_ to T_1_, no statistically significant differences were found (t_24_=–1.71; *P*=.09). However, the CG had a slightly higher mean increase in knowledge (mean 5, SD 3.68) compared to the EG (mean 3.2, SD 3.75). Analyzing the results based on question difficulty, the EG showed a significant knowledge increase for easy (*P*=.02), medium (*P*=.03) and hard (*P*<.001) questions. Similarly, the CG showed a significant knowledge increase for easy (*P*<.001) medium (*P*=.001) and hard (*P*<.001) questions. There were no significant differences between the groups in the knowledge increase for the questions of easy (t_24_=–0.68; *P*=.09) and medium (t_24_=–0.73; *P*=.09) difficulty. However, a significant difference was observed for hard questions (t_24_=0.64; *P*=.005), with the CG achieving a higher mean score (mean 5.76, SD 1.27) than the EG (mean 4.76, SD 1.39).

**Table 2:** Results for knowledge on home gardening.

Question	CG^a^				EG^b^				Intergroups, *P* value^c^
	T_0_^d^ scores, mean (SD)	T_1_^e^ scores, mean (SD)	*P* value^c^	T_0_ scores, mean (SD)		T_1_ scores, mean (SD)	*P* value^c^		
Easy (n=8)	4.28 (1.40)	5.68 (1.03)	<.001	4.04 (1.21)		5.08 (2.06)	.02	.09	
Medium (n=8)	3.64 (1.38)	4.80 (1.55)	.001	3.32 (1.77)		4.16 (1.62)	.03	.09	
Hard (n=7)	3.08 (1.32)	5.76 (1.27)	<.001	3.44 (1.23)		4.76 (1.39)	<.001	.005	
All (n=23)	11.12 (2.85)	16.12 (3.15)	<.001	10.88 (3.22)		14.08 (3.40)	<.001	.09	

^a^CG: control group.

^b^EG: experimental group.

^c^*P*<.05 was considered statistically significant.

^d^T_0_: before the intervention.

^e^T_1_: after the intervention.

### Attitude Toward Home Gardening

The attitude toward home gardening of participants (Table 3) in the EG significantly increased from T_0_ to T_1_ (mean difference 0.8, SD –0.01; *z*_24_=2.98; *P*=.003). When analyzing the results by question, significant differences (*P*<.05) were found for all items. In contrast, there was no significant difference in the attitude toward home gardening of the CG from T_0_ to T_1_ (mean difference –0.05, SD –0.09; *z*_24_=−0.54; *P*=.59) including individual questions. A significant difference was observed in attitude toward home gardening increase between groups from T_0_ to T_1_ (*z*_24_=2.73; *P*=.006). Furthermore, the EG exhibited a higher mean increase in attitude toward home gardening (mean 0.8, SD 1.21) than the CG (mean –0.05, SD 1.30).

**Table 3:** Results for attitude toward home gardening.

Question	CG^a^				EG^b^				Intergroups, *P* value^c^
	T_0_^d^ scores, mean (SD)	T_1_^e^ scores, mean (SD)	*P* value^c^	T_0_ scores, mean (SD)		T_1_ scores, mean (SD)	*P* value^c^		
1	4.44 (1.08)	4.52 (0.71)	.97	4.08 (1.19)		4.60 (1.07)	.04	—^f^	
2	4.20 (1.12)	4.40 (0.76)	.58	3.72 (1.21)		4.60 (1.12)	.009	—	
3	4.32 (0.94)	4.20 (0.87)	.56	3.56 (1.23)		4.48 (1.12)	.005	—	
4	4.32 (1.07)	3.96 (1.17)	.22	3.36 (1.22)		4.24 (1.16)	.008	—	
All	4.32 (0.93)	4.27 (0.84)	.59	3.68 (1.10)		4.48 (1.09)	.003	.006	

^a^CG: control group.

^b^EG: experimental group.

^c^*P*<.05 was considered statistically significant.

^d^T_0_: before the intervention.

^e^T_1_: after the intervention.

^f^Not applicable.

### Self-Efficacy in Home Gardening

The self-efficacy in home gardening of participants (Table 4) in the EG significantly increased from T_0_ to T_1_ (mean difference 0.75, SD –0.01; t_24_=2.6; *P*=.02). When analyzing the results by question, all items showed significant differences (*P*<.05). In contrast, the CG did not show a significant increase in self-efficacy in home gardening from T_0_ to T_1_ (mean difference –0.09, SD –0.17; t_24_=–0.36; *P*=.72) or in individual questions. Comparing the differences in self-efficacy in home gardening increase between the 2 groups revealed a significant difference (t_24_=2.21; *P*=.03). Furthermore, the EG exhibited a higher mean increase in self-efficacy in home gardening (mean 0.75, SD 1.45) than the CG (mean –0.09, SD 1.25).

**Table 4:** Results for self-efficacy in home gardening.

Question	CG^a^				EG^b^				Intergroups, *P* value^c^
	T_0_^d^ scores, mean (SD)	T_1_^e^ scores, mean (SD)	*P* value^c^	T_0_ scores, mean (SD)		T_1_ scores, mean (SD)	*P* value^c^		
1	3.68 (1.25)	3.84 (0.75)	.62	3.48 (0.87)		4.24 (0.97)	.02	—^f^	
2	3.60 (1.19)	3.60 (0.82)	.94	3.32 (0.90)		4.12 (0.97)	.02	—	
3	4.04 (1.14)	3.76 (0.88)	.22	3.52 (1.08)		4.32 (0.94)	.02	—	
4	4.00 (0.91)	3.76 (0.88)	.45	3.56 (1.16)		4.20 (0.87)	.03	—	
5	3.72 (1.02)	3.52 (1.00)	.53	3.60 (0.96)		4.24 (0.97)	.046	—	
6	3.64 (1.04)	3.64 (0.99)	.89	3.44 (1.23)		4.28 (1.02)	.02	—	
7	3.76 (1.01)	3.68 (0.90)	.78	3.44 (1.16)		4. 24 (0.88)	.03	—	
All	3.78 (0.93)	3.69 (0.76)	.72	3.48 (0.87)		4.23 (0.88)	.02	.03	

^a^CG: control group.

^b^EG: experimental group.

^c^*P*<.05 was considered statistically significant.

^d^T_0_: before the intervention.

^e^T_1_: after the intervention.

^f^Not applicable.

### Intention to Start a Home Garden

The intention to start a home garden of participants (Table 5) in the EG significantly increased from T_0_ to T_1_ (mean difference 2.02, SD 0; *z*_24_=4.15; *P*<.001). When analyzing the results by question, all items showed significant differences (*P*<.05). In contrast, the CG did not show a significant increase in intention to start a home garden from T_0_ to T_1_ (mean difference –0.18, SD 1.73; t_24_=–0.64; *P*=.53) or in individual questions. Comparing the differences in intention to start a home garden increase between the 2 groups revealed a significant difference (t_24_=–5.33; *P*<.001). Furthermore, the EG exhibited a higher mean increase in intention to start a home garden (mean 2.01, SD 1.46) than the CG (mean –0.19, SD 1.46).

**Table 5:** Results for intention to start a home garden.

Question	CG^a^				EG^b^				Intergroups, *P* value^c^
	T_0_^d^ scores, mean (SD)	T_1_^e^ scores, mean (SD)	*P* value^c^	T_0_ scores, mean (SD)		T_1_ scores, mean (SD)	*P* value^c^		
1	2.96 (0.84)	2.76 (1.42)	.52	2.00 (1.08)		4.04 (0.89)	<.001	—^f^	
2	3.04 (0.84)	2.72 (1.40)	.32	1.80 (0.91)		3.88 (1.09)	<.001	—	
3	3.04 (0.98)	3.00 (1.44)	.88	1.88 (1.05)		3.80 (1.15)	<.001	—	
All	3.01 (2.34)	2.83 (4.07)	.58	1.89 (0.95)		3.91 (0.95)	<.001	<.001	

^a^CG: control group.

^b^EG: experimental group.

^c^*P*<.05 was considered statistically significant.

^d^T_0_: before the intervention.

^e^T_1_: after the intervention.

^f^Not applicable.

### User Enjoyment

Results showed (Table 6) a significant difference between the participants’ answers and the neutral value in all categories: concentration (*z*_24_=4.11; *P*<.001; Cronbach α=0.90), goal clarity (*z*_24_=4.15; *P*<.001; Cronbach α=0.97), feedback (*z*_24_=3.92; *P*<.001; Cronbach α=0.96), challenge (*z*_24_=4.03; *P*<.001; Cronbach α=0.93), autonomy (*z*_24_=3.89; *P*<.001; Cronbach α=0.89), immersion (*z*_24_=3.83; *P*<.001; Cronbach α=0.93), social interaction (*z*_24_=3.82; *P*<.001; Cronbach α=0.93), and knowledge improvement (*z*_24_=3.92; *P*<.001; Cronbach α=0.96). All categories exhibited a Cronbach α of >0.70, which indicates a high internal consistency in the questionnaire [57]. These results state that users positively evaluated the video game in all categories.

**Table 6:** Results for user enjoyment.

Measure	Questions, N	T_1_^a^ score, mean (SD)	Neutral value	*P* value^b^	Range	Cronbach α
Concentration	6	4.45 (0.82)	3	<.001	1.67-5	0.90
Goal clarity	6	6.77 (0.81)	3	<.001	1-5	0.97
Feedback	5	4.60 (0.80)	3	<.001	1-5	0.96
Challenge	6	4.52 (0.79)	3	<.001	1.67-5	0.93
Autonomy	3	4.48 (0.84)	3	<.001	1-5	0.89
Immersion	7	4.34 (0.90)	3	<.001	1-5	0.93
Social interaction	6	4.26 (0.91)	3	<.001	1-5	0.93
Knowledge improvement	5	4.61 (0.81)	3	<.001	1-5	0.96

^a^T_1_: after the intervention.

^b^*P*<.05 was considered statistically significant.

### Relationship Between User Enjoyment and Efficacy Outcomes

Results showed (Table 7) no statically significant relationship between user enjoyment scores and posttest knowledge (EGameFlow scale: *r*_23_=0.26; *P*=.20) and intention (EGameFlow scale: *r*_23_=0.06; *P*=.77) scores. In addition, the results showed a positive statically significant relationship between user enjoyment scores and posttest attitude (EGameFlow scale: *r*_23_=0.72; *P*<.001) and self-efficacy (EGameFlow scale: *r*_23_=0.67; *P*<.001) scores.

**Table 7:** Correlations of user enjoyment scores with posttest efficacy results.

Category	*r*	*P* value^a^ (bilateral)
Player enjoyment and knowledge	0.26	.20
Player enjoyment and attitude	0.72	<.001
Player enjoyment and self-efficacy	0.67	<.001
Player enjoyment and intention	0.06	.77

^a^*P*<.05 was considered statistically significant.

### Hypothesis Evaluation

On the basis of the aforementioned results, in this subsection, we summarize the validation of each hypothesis using appropriate statistical methods. In addition, Table 8 summarizes the statistical significance, indicating whether each hypothesis was confirmed based on these results.

**Table 8:** Summary of hypothesis evaluation.

Hypothesis	Statistical analysis results	*P* value	Hypothesis confirmed?
1A	t_24_=4.26	<.001	Yes
1B	*z*_24_=2.98	.003	Yes
1C	t_24_=2.6	.02	Yes
1D	t_24_=2.21	.03	Yes
2A	t_24_=–1.71	.09	No
2B	*z*_24_=2.73	.006	Yes
2C	t_24_=2.21	.03	Yes
2D	t_24_=–5.33	<.001	Yes
3A	*r*_23_=0.26	.20	No
3B	*r*_23_=0.72	<.001	Yes
3C	*r*_23_=0.67	<.001	Yes
3D	*r*_23_=0.06	.77	No

## Discussion

### Principal Findings

#### Overview

The findings of this study support the effectiveness of Farmily in enhancing knowledge of home gardening, promoting a positive attitude toward home gardening, increasing self-efficacy in home gardening, and fostering the intention to start a home garden among novice individuals aged 20 to 50 years. In addition, Farmily demonstrated comparable effectiveness in knowledge transfer and a more significant impact on the other variables than a home gardening workshop facilitated by an expert in agriculture. Finally, Farmily yielded favorable results in user enjoyment, and we identified a significant positive relationship between user enjoyment and attitudes and self-efficacy and no relationship with knowledge and intention. The following sections comprehensively analyze each hypothesis, presenting the corresponding findings in detail.

#### Knowledge of Home Gardening

The EG demonstrated improved knowledge of home gardening after the treatment. Thus, our data support hypothesis 1A (confirmed). These results are consistent with those of previous studies on the effectiveness of video games in agricultural education [36-38,58]. Similarly, the CG also showed improved knowledge after the treatment. Regarding hypothesis 2A (rejected), there were no significant differences between the groups regarding the increase in knowledge overall and for easy and medium questions. However, a significant difference was found for difficult questions. These findings suggest that persuasive video games can be comparably effective to expert-led workshops in certain aspects but they may have limitations in more complex scenarios. It is important to acknowledge that workshops offer the advantage of direct interaction with experts, providing specific answers and in-depth knowledge. On the other hand, video games are constrained by the information provided within them. However, our video game demonstrated its potential effectiveness. Simulation-based experimentation has been successful in various fields, enabling competence development through trial-and-error experiences [59]. The discovery learning theory supports that knowledge is constructed through an iterative discovery process facilitated by educators [60]. Our results indicate that a serious persuasive video game can be valuable for reinforcing knowledge and complementing traditional educational techniques, particularly in noncomplex knowledge activities.

#### Attitude Toward Home Gardening

Regarding hypothesis 1B (confirmed), the participants in the EG demonstrated a significant improvement in their attitude toward home gardens. These results are consistent with findings of studies such as that on MahindiMaster [41], supporting our results. In contrast, the CG showed a nonsignificant decrease in attitude. Regarding hypothesis 2B (confirmed), the EG exhibited a higher mean increase in attitude than the CG. The divergence in results can be attributed to the unique firsthand experimentation experience provided by the simulation environment in Farmily. Actively engaging in virtual home gardening activities likely influenced participants’ perception of the garden’s interest, importance, and enjoyment, leading to a positive attitude toward real-life gardening. This finding aligns with the literature suggesting that providing new information can change attitudes toward an object [61]. The persuasive messages embedded in the video game may have also contributed to the favorable attitude change as persuasive technologies have been shown to influence attitudes and behaviors effectively [62,63].

#### Self-Efficacy in Home Gardening

Regarding hypothesis 1C (confirmed), participants in the EG demonstrated a significant improvement in their self-efficacy. These results are consistent with those of previous studies reporting positive effects on self-efficacy after intervention in video games [64]. Similarly, video games such as “Game of Piglets” [40] have increased players’ self-efficacy in agriculture-related topics following interventions, supporting our expectations for our video game. In contrast, the CG showed a nonsignificant decrease. Regarding hypothesis 2C (accepted), the EG exhibited a higher mean increase in self-efficacy than the CG. As described by Bandura [53], self-efficacy is an integral part of the self-system, encompassing a person’s attitude, ability, and cognitive abilities. According to Bandura [44], individuals develop self-efficacy beliefs through previous performance outcomes, vicarious experiences, social persuasion, and emotional and physiological states. The direct experience of EG users with the simulation of the home garden had a profound impact on their perception of their capabilities. The feedback and recommendation systems within the video game allowed players to analyze their previous performances, reinforcing their sense of competence. In addition, the recognition received when completing a level served as a form of social persuasion, offering congratulations and encouragement. In contrast, the workshop provided only vicarious experiences, lacking the opportunity for hands-on experimentation, which may explain its limited impact on self-efficacy.

#### Intention to Start a Home Garden

Regarding hypothesis 1D (confirmed), participants in the EG showed a significant improvement in their intention. These results were expected and explained through the Unified Theory of Behavior proposed by Jaccard et al [65], which states that 2 key factors influencing an individual’s intention to engage in a behavior are their attitude toward the behavior and their self-efficacy. In the case of the EG, attitude and self-efficacy showed significant increases, which directly contributed to enhancing their intention. Conversely, the CG did not exhibit significant differences in these variables, resulting in no notable change in intention. Regarding hypothesis 2D (confirmed), the EG exhibited a higher mean increase in intention than the CG. These results can also be explained by the UTAUT proposed by Venkatesh et al [55]. According to this theory, participants in the EG may have realized that the perceived complexity and effort associated with engaging in a home garden were lower than initially expected (expectation of effort). Moreover, their direct experimentation with the video game enabled them to discover their capabilities and potential for performing well in a real garden (performance expectancy). Furthermore, it is plausible that the video game impacted other variables such as social norms and emotions by including persuasive messages. However, it is important to note that no specific data or evidence regarding these phenomena were collected in the study. We did not identify previous studies on the intention to start a home garden, but given that a change in intention leads to a small to medium change in behavior [66], several studies that achieved a positive behavior change [21] may have successfully influenced intention. However, there is a need for more data to corroborate that assumption.

#### User Enjoyment

Farmily received highly positive ratings across various user enjoyment categories, including concentration, clarity of goals, feedback, challenge, autonomy, immersion, social interaction, and knowledge increase. Participants expressed satisfaction with the gameplay, level of challenge, clarity of objectives, and overall video game mechanics. The success of Farmily in providing positive user enjoyment can be attributed to the mixed methodology used during its design. The iterative development process and continuous feedback from home garden experts ensured the creation of a video game with a robust knowledge base. The interdisciplinary composition of the team, including a video game designer, an interaction expert, and an education expert, played a significant role in crafting clear, immersive, and enjoyable video game mechanics, pedagogical techniques, and interactive elements within the final product.

#### Relationship Between User Enjoyment and Efficacy Outcomes

Regarding hypotheses 3B (confirmed) and 3C (confirmed), the findings revealed a positive statistical relationship between user enjoyment scores and attitude and self-efficacy posttest scores. These results suggest that players who enjoy the game also have a positive attitude and can master the game. These results are consistent with those of previous studies on the relationship between enjoyment and attitudes (eg, environmental sustainability attitude [67] and attitude toward real-life refugees [68]) and the relationship between enjoyment and self-efficacy (eg, genocide awareness [69] and drinking refusal self-efficacy [70]). In addition, regarding hypotheses 3A (rejected) and 3D (rejected), there was no statistically significant correlation between user enjoyment scores and posttest scores measuring both knowledge and intention, suggesting that enjoyment does not appear to be associated with knowledge acquisition or the propensity to engage in home gardening. These results are particularly surprising given the contrast with previous research examining the connection between enjoyment and learning outcomes in serious gaming contexts, as evidenced by studies investigating food knowledge [71] or mathematical proficiency [72]. However, it is worth noting that some studies have also failed to establish a relationship between enjoyment and learning [73]. Furthermore, these findings diverge from those of studies exploring the relationship with behavioral intentions, such as those examining intentions related to reducing alcohol consumption [70].

### Limitations and Future Work

This study’s findings should be cautiously interpreted due to the small sample size (n=50). Future research would benefit from a larger sample to enhance statistical reliability. Despite this limitation, the results provide valuable insights into video games as a tool for promoting home gardening. The participant pool, composed solely of students and teachers, may not fully represent the broader adult population aged 20 to 50 years. Moreover, participants reported high previous experience with video games and technology, which may not represent the general population. The short treatment and evaluation period (90 minutes) only captures immediate effects, not longer-term impacts. In addition, the knowledge questionnaire was developed specifically for this study in collaboration with a home garden expert, potentially influencing the results. Finally, leveraging a serious video game for promoting home gardening in areas of a low socioeconomic status can empower individuals with practical skills, foster sustainability, and build community resilience. However, it must be implemented with careful consideration of literacy rates, technological access, cultural relevance, and ongoing engagement strategies to maximize its impact. An enhanced version of Farmily is planned, incorporating elements such as climate simulation, disease management, and crop rotation and association. Future investigations are underway to use a more diverse sample and extend follow-up periods, thereby enabling comprehensive analysis. Furthermore, these efforts seek to deepen our understanding of the intricate relationship between enjoyment and persuasive outcomes within gaming environments.

### Conclusions

Farmily, a 3D simulation single-player serious persuasive video game, was developed to educate and promote home gardening in novice individuals. The evaluation study demonstrated Farmily’s effectiveness in improving participants’ knowledge, attitude, self-efficacy, and intention to start their home gardens. Farmily demonstrated similar effectiveness in knowledge transfer to that of a traditional promotion workshop and had a more significant impact on other variables. The findings highlight Farmily’s potential to empower individuals and promote sustainable practices by teaching the required home gardening knowledge and persuading them to start a real home garden. Furthermore, player enjoyment substantially correlates with user attitudes and self-efficacy, yet no discernible association was observed between enjoyment and knowledge and intention. Future research could expand on the impact of Farmily by conducting studies with more extensive and diverse samples over an extended time frame, including a follow-up period. These studies would allow for a deeper understanding of the medium- and long-term effects of Farmily and the sustainability of its impact over time. Moreover, additional research endeavors have the potential to advance our knowledge of the intricate interplay between enjoyment and persuasive outcomes in gaming contexts.

## Appendix

Multimedia Appendix 1 CONSORT-eHEALTH checklist (V 1.6.1).

## Abbreviations

CG: control group
EG: experimental group
P-III: player-centered, iterative, interdisciplinary, and integrated
PSD: persuasive system design
RCT: randomized controlled trial
UTAUT: Unified Theory of Acceptance and Use of Technology
